# Technology of Informative Feature Selection for Immunosignature Analysis

**DOI:** 10.17691/stm2020.12.5.02

**Published:** 2020-10-28

**Authors:** A.A. Koshechkin, O.V. Romanovich, D. Stamate, S.A. Johnston, A.V. Zamyatin

**Affiliations:** Assistant, Department of Theoretical Foundations of Informatics; National Research Tomsk State University, 36 Lenin Avenue, Tomsk, 634050, Russia;; Associate Professor, Department of Theoretical Foundations of Informatics; National Research Tomsk State University, 36 Lenin Avenue, Tomsk, 634050, Russia; Leading Engineer, Institute of Applied Mathematics and Computer Science; National Research Tomsk State University, 36 Lenin Avenue, Tomsk, 634050, Russia;; Senior Lecturer; Data Science Department of Computing, Goldsmiths, University of London, New Cross, London, SE14 6NW, UK;; Center Director and Professor; Biodesign Center for Innovations in Medicine, Arizona State University, Tempe, AZ 85281, USA; Head of the Department of Theoretical Foundations of Informatics; National Research Tomsk State University, 36 Lenin Avenue, Tomsk, 634050, Russia; Director of the Institute of Applied Mathematics and Computer Science National Research Tomsk State University, 36 Lenin Avenue, Tomsk, 634050, Russia;

**Keywords:** early diagnosis of diseases, immunosignature, feature selection in the sample, machine learning

## Abstract

The main difficulty in practical work with data obtained via immunosignature analysis is high dimensionality and the presence of a significant number of uninformative or false-informative features due to the specific character of the technology. To ensure practically relevant quality of data analysis and classification, it is necessary to take due account of this specific character.

**The aim of the study** is to create and test the technology for effective reduction of immunosignature data dimensionality, which provides practically relevant and high quality of classification with due regard for the properties of the data obtained.

**Materials and Methods.:**

The study involved the use of two normalized data sets obtained from the public biomedical repository and containing the results of immunosignature analysis.

The technology for selecting informative features was proposed within the framework of the study. It consisted of three successive steps: 1) breaking a multiclass task into a series of binary tasks using the “one vs all” strategy; 2) screening of false-informative features is performed for each binary comparison by comparing the values of the median of the sets “one” and “all”; 3) ranking of the remaining features according to their informative value and selection of the most informative ones for each binary comparison.

To assess the quality of the proposed technology for informative feature selection, we used the results obtained after application of classification based on the filtered data. Support vector method that proved itself in the problems of high-dimensional data classification was used as a classification model.

**Results.:**

Effectiveness of the proposed technology for informative feature selection was determined. This technology allows us to provide high quality of classification while significantly reducing the feature space. The number of features eliminated in the second step is approximately 50% for each data set under consideration, which greatly simplifies subsequent data analysis. After the third step, when the feature space is reduced to 15 features, the quality of classification by the macro-average F1-score metric is assessed as 98.9% for the GSE52581 dataset. For the GSE52581 dataset, with the feature space reduced to 266 features, the quality of classification by the macro-average F1-score metric is 91.3%.

**Conclusion.:**

The results of the work demonstrate the promising outlook of the proposed technology for informative feature selection as applied to the data of immunosignature analysis.

## Introduction

In 2018, in Russia there were 624 thousand patients who were diagnosed with an oncological disease for the first time in their lives: of them, 30.6% had stage I, 25.8% — stage II, 18.2% — stage III, 20.3% — stage IV. In Russia, cancer mortality amounted to more than 293 thousand people in 2018. At the same time, no statistically significant changes in the absolute number of deaths due to malignant neoplasms have been observed over the past 5 years [1].

Cancer treatment efficacy directly depends on timely diagnosis. Early detection of cancer requires effective and patient-specific, easy-to-use, patient-friendly, and inexpensive diagnostic methods [2]. The technology of immunosignature analysis based on the idea of human antibody profiling is one of the most promising methods [3]. This technology is based on a microarray, which is a set of peptides with random amino acid sequences that provide a map of immune activity when interacting with human blood serum. There is a wide variety of peptide arrays containing 10 thousand to 330 thousand peptides.

At present, the applicability of various data mining and classification methods for the analysis and interpretation of data obtained via immunosignature analysis is actively studied. To build effective classification models, researchers need relevant and high quality data. The feature space is based on randomly created peptides, therefore not all features are likely to be informative; hence, their selection is one of the most important stages of data analysis. Discarding useless and redundant features not only improves model performance but also facilitates its interpretation [4]. In this regard, each article devoted to investigation of immunosignature data is bound to pay special attention to the stage of selecting informative features.

Work [5] dwells on the applicability of immunosignature analysis for detecting four different pancreatic diseases (cancer and precancerous condition, type 2 diabetes, and pancreatitis). At an early stage, these diseases have similar symptoms, which complicates the diagnosis. The authors used the Student’s t-test to select the best features for further analysis. The average classification accuracy amounted to 92%. At the same time, each disease was found to have unique immunological characteristics.

The authors of study [6] demonstrate that the technology of immunosignature analysis has the potential to meet the requirements of a universal test for cancer diagnosis. An intellectual analysis of two data sets of the 6^th^ and 15^th^ grades was carried out. As a result, it was experimentally shown that immunosignature analysis makes it possible to separate different types of diseases with high precision. U-test was used to select informative features.

Work [7] investigates the possibilities of using the technology of immunosignature analysis using the example of a microarray with 330 thousand peptides for diagnosing breast cancer. The main idea of the study was to use the method of Projection to Latent Structures to identify effective data dimensionality. This was supposed to reduce the negative effect of model overfitting and improve object recognition quality. This approach goes against the main idea of immunosignature analysis aimed at finding possible antigens for various diseases. On applying the method of Projection to Latent Structures, the initial feature space is transformed into a new space of latent structures. In this regard, it becomes impossible to interpret the feature space in the context of “antigen–antibody” interaction. U-test for selecting the best features, also used by the researchers from the previous article, is considered to be alternative to Projection to Latent Structures.

The use of statistical criteria for informative feature selection is an example of using the filtering methods. These methods are characterized by such problems as non-obviousness of the threshold for separating uninformative features and preservation of feature space redundancy. Analysis of data with redundant features generally requires a lot of memory and computing power, and can also cause such undesirable effect as classification model overfitting [8]. At the same time, the origin of data is not taken into account, which can result in non-obvious errors.

**The aim of the study** is to create and test the technology for effective reduction of immunosignature data dimensionality, which provides practically relevant and high quality of classification.

## Materials and Methods

The study used two normalized data sets (GSE52580 and GSE52581) obtained from the public biomedical repository and containing the results of immunosignature analysis in patients with various oncological and infectious diseases as well as healthy individuals representing the control group [9, 10]. Previously, the datasets were transposed to correspond to the tidy data format [11]. The resulting materials are a set of data (a table) of peptide fluorescence intensity values, where peptide names are columns (features), and class labels are rows (samples).

The GSE52580 dataset has the following characteristics:

the number of samples — 240;

the number of features — 9787;

the number of classes — 6.

The number of each class samples is the same in the GSE52580 dataset.

The GSE52581 dataset has the following characteristics:

the number of samples — 1516;

the number of features — 10,372;

the number of classes — 15.

The number of each class samples is different in this set (Table 1).

**Table 1:** Description of data set GSE52581

Class	The number of samples
Healthy	249
Astrocytoma	166
Coccidioidomycosis	142
Breast cancer	141
Pancreatic cancer	136
Multiple myeloma	112
Lung cancer	107
Mixed oligoastrocytoma	97
Ovarian carcinoma	86
Pancreatitis	82
Recurrent breast cancer	61
Oligodendroglioma	48
Stage IV breast cancer	42
Glioblastoma multiforme	27
Ewing’s sarcoma	20

### Technology description.

 The technology for informative feature selection was proposed within the framework of the study. It consisted of three successive steps:

1. breaking a multiclass task into a series of binary tasks using the “one vs all” strategy;
2. screening of false-informative features is performed for each binary comparison by comparing the values of the median of sets “one” and “all”;
3. ranking of the remaining features according to their informative value and selection of the most informative ones for each binary comparison.

*The first stage of the technology is the application of the “one vs all” strategy.* In order to select informative features, multiclass tasks are broken into several binary tasks using the “one vs all” or “one vs one” strategy with subsequent selection of the best features for each binary comparison [12]. This study, we use “one vs all” strategy. This decision stems from the special aspect of immunosignature analysis technology consisting in imitation of disease antigens using random amino acid sequence peptides. The purpose of the analysis at this stage is to find a peptide that plays the role of an antigen for a specific disease, i.e. antibodies produced against the given disease bind to this peptide, while this does not occur in patients from groups with a different diagnosis.

*The second stage of the technology is the median filter.* Filtering methods are feature ranking methods that assess feature relevance by considering the intrinsic data properties [13]. After ranking, the features with informative value estimated as lower than the threshold value are removed. The resulting subset of features is used for further data analysis (for example, classification via machine learning methods). Filtering methods are easily scalable for high-dimensional data and have low computational complexity, but most of them are applicable only to binary tasks considering each feature separately and ignoring feature dependencies, which can negatively affect subsequent data analysis.

**Figure 1:**
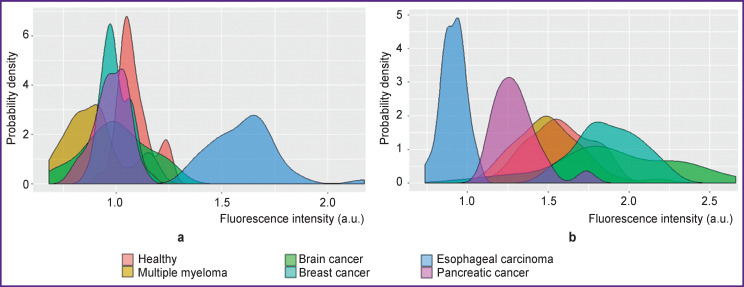
Comparison of informative (peptide CSGTMNSEFQNTTRHVYIMS) (a) and false-informative (peptide CSGVFMLSHHQFHPSWYQPN) (b) features for the class “esophageal cancer”

The first filter is a comparison of the median values for each feature for the set “one” and the set “all”. The aim of this filter is to remove from the dataset all features for which the median fluorescence intensity value of set “one” is less than the median fluorescence intensity of set “all” as they are considered to be uninformative. The reason for this lack of information value is illustrated by the example of two features from the GSE52580 dataset (Figure 1).

If we look at these two features in isolation from the subject area, both of them will be informative for separating the class “esophageal cancer” from all the others. However, when distribution of fluorescence intensity of the class “esophageal cancer” is to the right of all other classes, this means that antibodies from the blood of patients diagnosed with esophageal cancer bound to the peptide in a greater number than in other classes presented (Figure 1 (a)). The case, when fluorescence intensity distribution is to the left of all other classes, implies that this peptide bound to antibodies of all classes, including “healthy” ones, but excluding “esophageal cancer” (Figure 1 (b)). This means that there have been recorded antibodies developed for some other disease somehow uniting people of these groups, but unrelated to the diagnosis of esophageal cancer. All the earlier studies devoted to immunosignature analysis that we reviewed did not cover this aspect in any way and both variants of features were considered as informative. In turn, there are actually no informative features for objects of the class “healthy”; in fact, these are exceptions that did not fall into any other class.

*The third stage of the technology is ranking and selection of features.* The criterion of symmetric uncertainty (SU) is used as the second filter for estimating features. This criterion for estimating the correlation between both the features and the target variable is an improved version of the information gain criterion [14]. SU values are in the range [0; 1], where 0 means the complete absence of correlation and, consequently, irrelevance of the feature.


$$SU(X;\text{​ }Y)\;=\;\frac{2\cdot IG(X;\text{​ }Y)}{H(X)+H(Y)},$$

where *IG*(*X*; *Y*)=*H*(*X*)–*X*(*X*|*Y*) — the information gain for the feature and label of class *Y*; *H*(*X*) — the entropy of feature *X*; *H*(*Y*) — the entropy of feature *Y*.

The next step is to select a subset of informative features. For this purpose, the best features are selected for each binary comparison based on the estimates by the SU method.

### Evaluation of informative feature selection efficiency.

 The main goal of immunosignature analysis is the diagnosis of diseases, which in terms of data analysis is a classification task. In this regard, to assess the quality of the proposed technology for informative feature selection, we used the results of classification obtained after its application.

There are many different classification methods that can be used to accomplish this task. Let us look at the machine learning technique that has already proven its high efficiency in previous studies [15], the support vector machine (SVM), which is based on the construction of a hyperplane maximally separating the classes [16]. Depending on the kernel settings, it is possible to build separating hyperplanes of various kinds. There is no general approach to automatic kernel selection, so this study evaluates efficiency of each of them.

In addition, it is necessary to standardize features in linear models (for example, SVM) due to the following circumstance. One of the most important assumptions when working with linear models whose parameters are estimated by the least squares method is that the residuals of the model are independent (i.e. not correlated) and have normal distribution with the mean value of 0 and some fixed standard deviation (for example, 1). Therefore, the features were standardized in this study according to the following formula:


$$Z=\frac{X_{i}-\bar{X}}{\sigma },$$

where *X_i_* is an individual value for the feature; *X*¯ — mean value for the feature; σ — standard deviation for the feature.

There are many different classification quality assessment metrics that are suitable for the presented task. In this study, we use precision, recall, balanced accuracy, and F1-score [17] due to the objectives of the analysis: it is necessary to effectively separate the group of persons with a certain disease from all others, and the presented metrics make the separation possible. These will be computed for each “one vs all” binary comparison, where “one” is a positive class and “all” is a negative class.

### Experimental research.

 Due to the small number of samples in the dataset, cross-validation analysis is required. Thus, the original dataset is divided into 5 approximately equal parts, observing the proportion of classes. At each iteration, 4 parts form a training set, while the 5^th^ part forms a test set with a subsequent change. At each iteration, informative features are found based on training and assessed using the test set. The following is a description of one cross-validation iteration.

1. Evaluation of the informative value of features in the training set using the SU method and applying the “one vs all” strategy.
2. Conversion of false-informative feature estimates to 0 based on the filtering results and comparison of medians.
3. Selection of the best features for each binary comparison (N) until acceptable classification results are achieved.
4. SVM training of the training set, based on the obtained subset of features, and evaluating the classification quality using the test set.
5. Evaluation of the results obtained.

The work was done using the R programming language and libraries available at the CRAN and Bioconductor public repositories.

## Results

**Figure 2:**
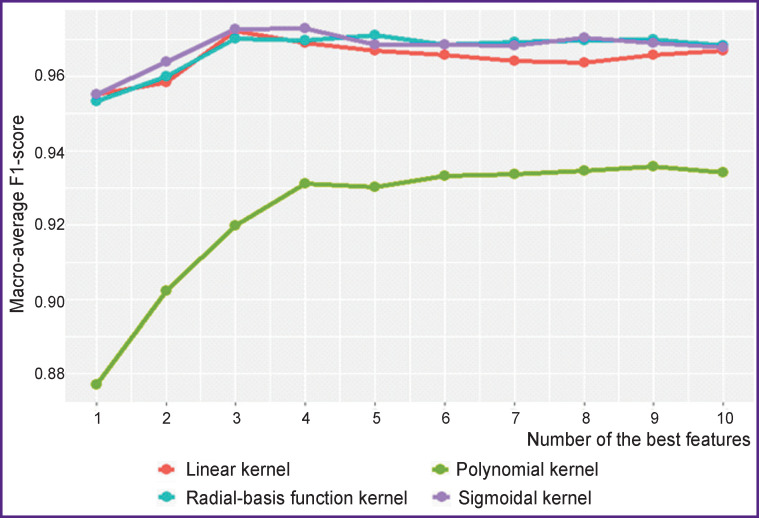
Classification results for various SVM kernels depending on the number of the best features (the GSE52580 set)

First of all, let us look at the results of experiments on evaluating the efficiency of various SVM kernels and selecting informative features with the proposed technology for the GSE52580 dataset. Figure 2 shows the classification results for each SVM kernel according to the macro-average F1-score metric.

Information in Figure 2 demonstrates that the best results are shown by the linear, sigmoidal, and radial-basis kernels, though the linear kernel is much superior to its analogs in performance. In this regard, only the linear kernel will be used further.

**Figure 3:**
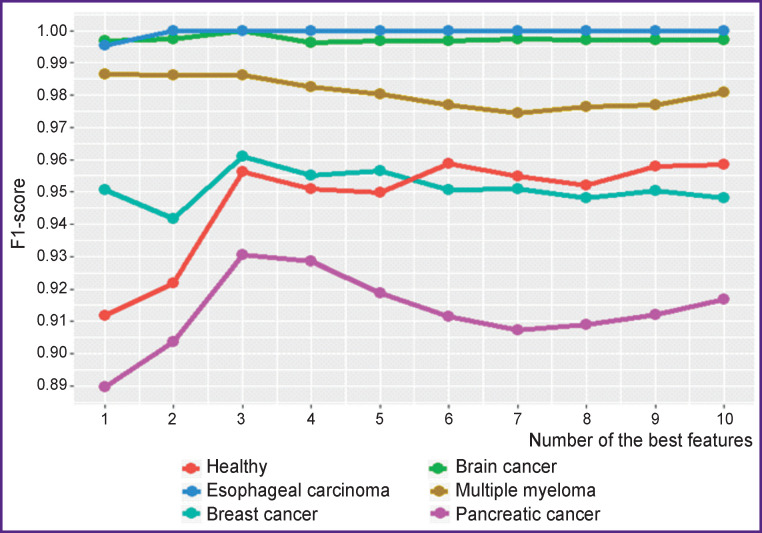
Classification results for each class depending on the number of the best features (the GSE52580 set)

Figure 3 shows the results of classification with SVM (linear kernel) using the F1-score metric, depending on the number of selected best features for each class.

The figure clearly shows that the best results are achieved when selecting the best three features for each class, except for “healthy” ones (there are no informative features for the “healthy” class objects, in fact, these are exceptions that did not fall into any other class), which means a total of 15 features for 6 classes.

Table 2 shows the results of classification on the test set using only the best three features for each disease. The values in the table are various quality assessment metrics, averaged when doing cross-validation. The ratings of the classification quality presented in the table indicate separability of classes, despite the significant reduction in the feature space.

**Table 2:** The results of classification on the test sample based on the best three features for each disease

Class	Metrics			
	Precision	Recall	F1-score	Balanced accuracy
Brain cancer	1	1	1	1
Breast cancer	0.976	1	0.988	0.997
Esophageal cancer	1	1	1	1
Healthy	0.969	0.993	0.981	0.993
Multiple myeloma	1	0.975	0.987	0.987
Pancreatic cancer	0.968	0.944	0.956	0.969

**Figure 4:**
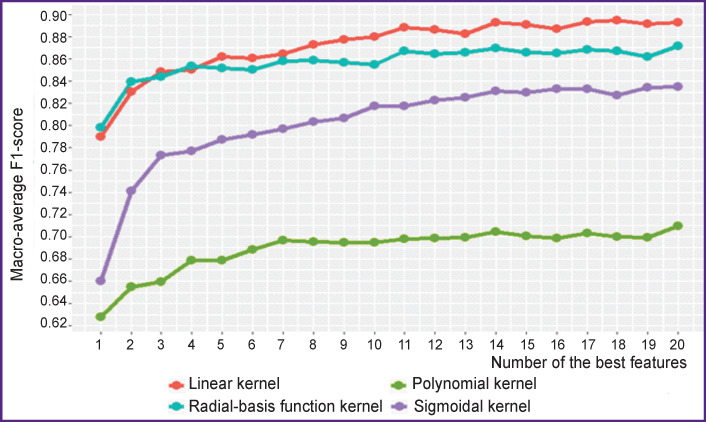
Classification results for various SVM kernels depending on the number of the best features (the GSE52581 set)

Now, let us look at the results of experiments on evaluating the efficiency of various SVM kernels and selecting informative features using the proposed technology for the GSE52581 dataset. Figure 4 shows the results of classification using the macro-average F1-score metric for each SVM kernel.

The figure clearly shows the advantage of the linear kernel over others; therefore, only this kernel is used in the further analysis of this set.

**Figure 5:**
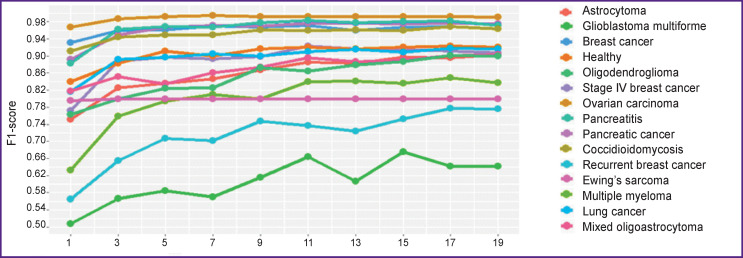
Classification results for each class depending on the number of the best features (the GSE52581 set)

Figure 5 shows the results of classification with the F1-score metric, depending on the number of selected best features for each class.

Table 3 shows the results of classification on the test set using only 19 best features for each class, except for the “healthy” (there are no informative features for the “healthy” class objects, in fact, these are exceptions that did not fall into any other class). The values in the table are various quality assessment metrics, averaged when doing cross-validation. It is clearly seen that high classification quality has been achieved for the vast majority of classes, despite the significant reduction in the feature space.

**Table 3:** The results of classification on the test sample based on 19 best features for each disease

Class	Metrics			
	Precision	Recall	Metrics F1-score	Balanced accuracy
Astrocytoma	0.915	0.903	0.908	0.946
Breast cancer	0.971	0.979	0.975	0.988
Stage IV breast cancer	0.915	0.978	0.942	0.988
Glioblastoma multiforme	0.756	0.767	0.738	0.881
Healthy	0.893	0.968	0.928	0.972
Lung cancer	0.867	0.963	0.912	0.976
Mixed oligoastrocytoma	0.99	0.865	0.922	0.932
Multiple myeloma	0.962	0.757	0.84	0.877
Oligodendroglioma	0.938	0.85	0.887	0.924
Ovarian carcinoma	0.989	0.989	0.989	0.994
Pancreatic cancer	0.958	0.991	0.974	0.993
Pancreatitis	0.973	0.962	0.967	0.980
Recurrent breast cancer	0.786	0.771	0.768	0.881
Ewing’s sarcoma	1	1	1	1
Coccidioidomycosis	0.964	0.956	0.960	0.976

The obtained classification results are consistent with previous studies in this field. However, the key aspect of the work is the absence of false-informative features in the final feature space, which was not given due regard earlier. This will produce a positive effect on the subsequent analysis and identification of antigens for various diseases.

## Conclusion

The main difficulty in practical work with data obtained via immunosignature analysis is high dimensionality and the presence of a significant number of uninformative or false-informative features due to the specific character of the technology. To ensure practically relevant quality of data analysis and classification, it is necessary to take due account of this specific character. The proposed technology for informative feature selection provides high estimates of classification quality while significantly reducing the feature space.

The number of features eliminated in the second step is approximately 50% for each data set under study, which greatly simplifies subsequent data analysis. After the third step, when the feature space is reduced to 15 features, the quality of classification by the macro-average F1-score metric is assessed as 98.9% for the GSE52580 dataset. For the GSE52581 dataset, with the feature space reduced to 266 features, the quality of classification by the macro-average F1-score metric is 91.3%.

The results of the investigation demonstrate the promising outlook of the proposed technology for informative feature selection as applied to the data of immunosignature analysis.
